# Web-based visual analysis for high-throughput genomics

**DOI:** 10.1186/1471-2164-14-397

**Published:** 2013-06-13

**Authors:** Jeremy Goecks, Carl Eberhard, Tomithy Too, The Galaxy Team, Anton Nekrutenko, James Taylor

**Affiliations:** 1 Department of Biology, Emory University, 1510 Clifton Road NE, Atlanta, GA 30322, USA; 2 Department of Mathematics and Computer Science, Emory University, 400 Downman Dr., Atlanta, GA 30322, USA; 3 Department of Biological Sciences, National University of Singapore, 14 Science Drive 4, 117543, Singapore; 4 Center for Comparative Genomics and Bioinformatics, Penn State University, 505 Wartik Lab, University Park, PA 16802, USA

**Keywords:** Visualization, Visual analysis, Galaxy, Genome browser, Circos, Phylogenetic tree

## Abstract

**Background:**

Visualization plays an essential role in genomics research by making it possible to observe correlations and trends in large datasets as well as communicate findings to others. Visual analysis, which combines visualization with analysis tools to enable seamless use of both approaches for scientific investigation, offers a powerful method for performing complex genomic analyses. However, there are numerous challenges that arise when creating rich, interactive Web-based visualizations/visual analysis applications for high-throughput genomics. These challenges include managing data flow from Web server to Web browser, integrating analysis tools and visualizations, and sharing visualizations with colleagues.

**Results:**

We have created a platform simplifies the creation of Web-based visualization/visual analysis applications for high-throughput genomics. This platform provides components that make it simple to efficiently query very large datasets, draw common representations of genomic data, integrate with analysis tools, and share or publish fully interactive visualizations. Using this platform, we have created a Circos-style genome-wide viewer, a generic scatter plot for correlation analysis, an interactive phylogenetic tree, a scalable genome browser for next-generation sequencing data, and an application for systematically exploring tool parameter spaces to find good parameter values. All visualizations are interactive and fully customizable. The platform is integrated with the Galaxy (http://galaxyproject.org) genomics workbench, making it easy to integrate new visual applications into Galaxy.

**Conclusions:**

Visualization and visual analysis play an important role in high-throughput genomics experiments, and approaches are needed to make it easier to create applications for these activities. Our framework provides a foundation for creating Web-based visualizations and integrating them into Galaxy. Finally, the visualizations we have created using the framework are useful tools for high-throughput genomics experiments.

## Background

Visualization plays an integral role in scientific investigation; it is useful for viewing large amounts of data simultaneously, observing patterns and outliers amongst data, and communicating findings to others. Traditionally, visualization has been the final step in a genomic experiment, used to view the results of a multi-step workflow. However, this approach limits the usefulness of visualization because, should viewing the results reveal something unexpected, the problem must be diagnosed and complete workflow rerun. An alternative and powerful approach is to combine visualization with analysis tools to perform visual analysis. In visual analysis, visualization and tools are blended together to enable seamless—and often integrative use—of both to understand data, try different approaches, and diagnose issues.

Visual analysis can simplify the use and creation of analysis pipelines. When using a pipeline with multiple tools, it is often useful to be able to check the data produced by each tool to ensure that it is reasonable. Without visual analysis, data must be downloaded, the visualization software opened, and only then can the data be loaded and visualized. If tools and visualization are integrated, however, a single button can be used to view the data; once the data has been validated, the pipeline can be resumed. Switching between visualizing data and running analysis tools is useful for more complex tasks as well. For example, in tool parameter space exploration, it’s useful to be able to run a tool with many different settings and use visually compare tool outputs for different settings.

There is growing consensus that visual analysis is needed for high-throughput genomic workflows and experiments [1]. Integrating a limited set of analysis tools with genome visualizations was the first step taken towards visual analysis. For example, BLAT searches can be run and then immediately visualized in the UCSC browser [2], the IGV genome browser includes filters for dynamically filtering tracks based on metadata attributes [3], the Artemis browser includes real-time SNP filtering as well as simple calculations (e.g., read density, expression level) for small datasets [4], the Spark tool groups and display similar genomics regions together in real time [5], and the StratomeX application supports interactive clustering of genomic data to identify potential relationships amongst clusters [6]. This approach is limited, though, as many visualizations are closely coupled with tools, making it difficult to incorporate new tools. The Savant platform addresses this limitation by providing a platform that supports analysis plugins. Savant includes multiple modes as well as a plugin framework for developing analysis tools such as SNP calling algorithms and dynamic [7,8]. Similarly, taken together, Bioconductor [9] can be considered a visual analysis platform because it includes modules for both analysis and visualization. Finally, a challenge with all visual analysis approaches is that many tools run for hours or days to operate on genome-scale data, making them impractical for interactive use.

Drawing inspiration from this prior work, we have developed a framework for creating Web-based visualizations and visual analysis applications. The Web is ideal for visualization/visual analysis because data can be used remotely rather than downloading it, a significant advantage because high-throughput genomics data is very large. In addition, the Web is useful for sharing visualizations with colleagues because the only software required is a Web browser, which everyone has. Our framework for doing visual analysis on the Web provides (a) client-side and server-side components for visualizing genomic data and (b) integration with the popular online genomics workbench Galaxy (http://galaxyproject.org) [10,11]. The framework’s components help manage data flow between Web browser and Web server, provide methods for indexing and quickly obtaining data from large genomic datasets, integrate visualization and analysis tools, and enable sharing and publication of saved visualizations. By integrating with Galaxy, the framework enables any Web-based visualization to use Galaxy tools and integrate with Galaxy’s analysis workspace.

Using our framework, we have developed and integrated numerous visual applications into Galaxy. These include a Circos-style genome-wide viewer, an interactive phylogenetic tree, a generic scatter plot application, a genome browser, and an application for visually finding good parameter values for analysis tools. These applications support visual analysis through the use of analysis tools and user interaction/customization. While all the applications discussed enable some degree of visual analysis, we refer to each of them as a ‘visualization’ or ‘visual application’ for simplicity.

## Implementation

Enabling Web-based visual analysis required two implementation efforts. First, we developed a collection of client and server components that provide common, reusable building blocks for creating genomic visualizations on the Web. Next, we integrated those components into Galaxy (http://galaxyproject.org) [10,11] to take advantage of Galaxy’s features and, ultimately, build more powerful visual applications.

### Components for building Web-based visualizations

A library of JavaScript objects comprise the framework’s client (Web browser) components. Some objects are applicable to all visualizations, such as a base Visualization object for easily creating and saving applications and a Cache object for storing items, especially data. Many objects are specific to visualizations that use genomic data. These objects include: (1) a GenomeDataManager that requests, organizes, and stores data obtained from the server; (2) Track objects that denote genomic datasets; (3) Genome objects that include information such as chromosome lengths; and (4) Bookmark objects for genomic regions with annotations. Using only JavaScript and HTML to build an application ensures that only a Web browser is needed to use it.

The framework’s server components include data converters, indexers, and providers written in Python. Converters and indexers transform or augment a dataset so that it can be efficiently queried for data. Visualizations only need retrieve and display data in the region or area being viewed, so indices are critical because they can help provide data quickly. Data providers use indices to return data requested by applications. There are often multiple data providers for a data type so that both summary (e.g., coverage) and detailed data can be provided. Providing both summary and detailed data is important because it is often not feasible to return all the individual data points for a large region. Trying to get all mapped reads for a chromosome, for instance, could yield millions of reads, which is too much data to provide to a Web visualization. Instead, data providers produce coverage data when there are many reads or features in a region, and individual reads/features are produced when a smaller region is queried. Our framework includes data providers for many common genomic formats, including SAM/BAM, BED, Interval, GFF/GTF, VCF, BedGraph, Wiggle, and BigWig/BigBed. There are also data providers for tabular and phylogenetic data. Using data providers to query datasets is done through a RESTful API.

### Integration with Galaxy

We have integrated our framework’s components into Galaxy (http://galaxyproject.org). Galaxy is a open, Web-based platform that can be used for all facets of genomic analyses, including data retrieval and integration from popular databases, multi-step analysis, repeated analyses via workflows, collaboration, and publication. Integration with Galaxy amplifies the value of Web-based visualizations/visual analysis applications because they can be used together with Galaxy’s other features. Applications benefit substantially by virtue of access to Galaxy’s large collection of analysis tools. Integrating our framework’s components extends Galaxy to support Web-based visualizations. If existing data converters, data providers, and client objects are used, integrating a visualization into Galaxy is as simple as writing the application in HTML and JavaScript— often using framework JavaScript components—and registering it with Galaxy. Galaxy’s visualization framework, then, is flexible enough to accommodate nearly any Web-based visual application.

Visualizations can be opened in Galaxy’s analysis workspace via icons associated with a dataset. Because the data is stored in Galaxy, no downloading or formatting is necessary to use datasets in Galaxy visual applications. Visualizations can be saved and shared with individuals or published via URL using the Galaxy publishing framework, making them ideal for including in supplementary materials. Shared or published visualizations are fully functional and can be copied and modified. Galaxy visual applications require only a modern Web browser to use; no programming skills are needed. Our framework enables anyone to create Web-based visualizations and integrate them into Galaxy.

## Results and discussion

Using our framework, we have integrated both stand-alone visualizations as well as complex visual analysis applications into Galaxy. Visualizations include a scatter plot, a phylogenetic tree, and a Circos-style [12], genome-wide viewer. The visual analysis applications directly incorporate Galaxy tools. One application is a genome browser for high-throughput sequencing data in which tools can be run and their output visualized immediately. The other application helps identify good parameter settings for a tool via systematic exploration of a tool’s parameter space. These visualizations and visual analysis applications leverage Web technologies to make them highly interactive and customizable, yet they require require no dataset downloads and no software beyond a Web browser. Customization and interactivity during visual analysis is important because high-throughput sequencing data, due to its low cost and high fidelity, is being used in increasingly diverse biomedical experiments. For visual analysis to be effective in a variety of contexts, it must be able to adapt visual analysis tools as needed.

### Scatterplot

**Figure 1:**
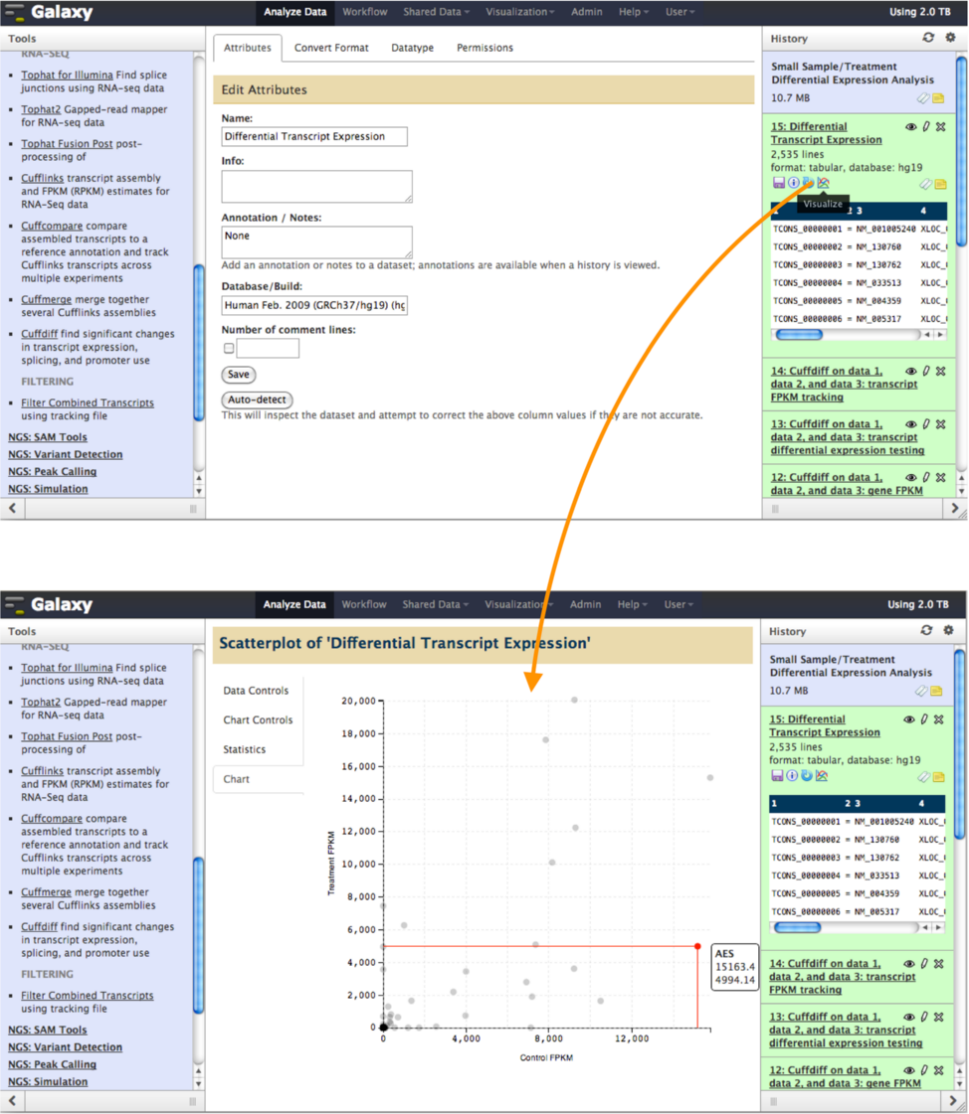
**Galaxy Scatterplot visualization of transcript differential expression from the Tophat-Cufflinks pipeline for an experiment measuring expression of ~2,500 transcripts in brain and adrenal tissue from the BodyMap 2.0 dataset (EBI accession E-MTAB-513).** Clicking on the visualize icon for the dataset (top) opens up the scatter plot visualization directly in Galaxy’s analysis workspace (bottom). Columns for control and treatment FPKM are visualized, and gene name is used for labeling. The scatterplot shows that most transcripts are expressed at low levels in both conditions. However, a small number of transcripts are highly expressed in either the control, treatment, or both. Hovering over a data points shows its label and data; a transcript for the AES gene shows decreased expression in the treatment condition as compared to the control condition.

Tabular data is common in genomics experiments, and the Scatterplot visualization makes it easy to create interactive scatter plots for columns of numerical data (Figure 1). Creating a scatter plot for a dataset can be done in second by clicking on the dataset’s visualize icon and choosing the columns to use for the plot’s data. The plot derives minimum and maximum values for axes from the data and includes grid lines for reference. The plot can be customized on the fly by adding an id value from another column to each datapoint, setting axis labels and datapoint sizes, and plot width and height. Hovering over an individual data point shows its label and values, and statistics for the plot’s data are available as well. Scatter plots are displayed in the main window of Galaxy’s analysis interface, making it possible to do visual analysis by toggling between running analysis tools and generating plots of output datasets. One common use of a scatter plot in genomics is visualizing differential expression data. Galaxy includes the Tophat-Cufflinks pipeline [13] for doing differential expression using RNA-seq data, and Figure 1 shows a scatterplot of transcript expression between two different human tissues.

### Phylogenetic tree

**Figure 2:**
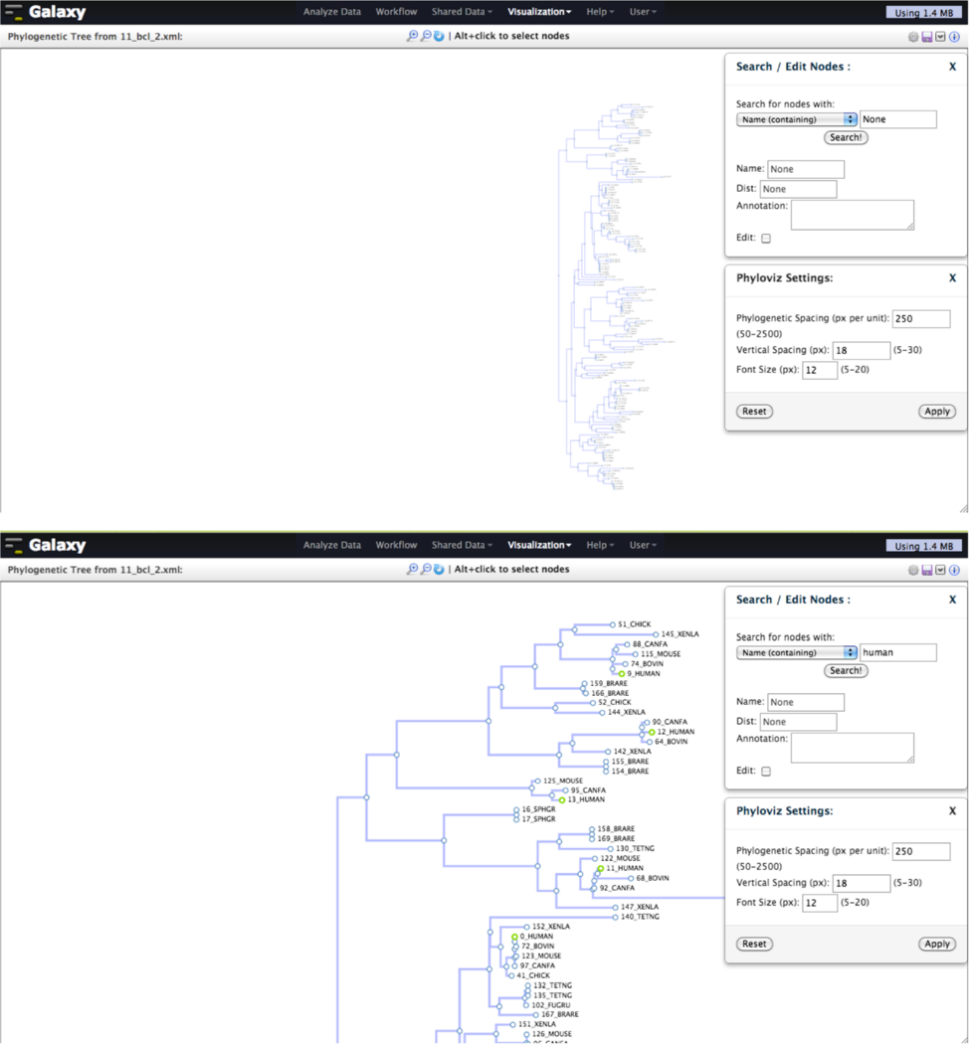
**Galaxy PhyloViz visualization for the BCL2 gene phylogenetic tree across species; the BCL2 gene tree is a canonical tree for the PhyloXML tree format and is available at **http://www.phyloxml.org/**.** Top: the entire tree is visible when zoomed out; Bottom: zoomed in on the top half of the tree and, using search, nodes with human genes have been highlighted in green. All actions—e.g., zooming, searching—are done in the Web browser and no page refreshes are necessary.

PhyloViz is an interactive viewer for large phylogenetic trees (up to ~10,000 nodes) that provides powerful navigation and editing capabilities (Figure 2). All three popular phylogenetic formats (PhyloXML, Newick, and Nexus) can be visualized in PhyloViz. There are other software packages for creating interactive phylogenetic trees on the Web [14-16], but PhyloViz takes advantage of new Web technologies to provide unique features. PhyloViz uses D3 [17] for fast, efficient rendering of large trees in SVG format and for leveraging graphical transitions to make tree navigation and editing more intuitive. PhyloViz supports panning and zooming to navigate around trees, and subtrees can be expanded or collapsed by clicking on individual nodes. Using PhyloViz, tree attributes can be edited: nodes can be annotated and node-node distances can be changed; tree display parameters, such as font size and vertical/horizontal spacing can be adjusted in order to customize the tree’s display as needed. Finally, nodes can be searched by name, annotation, or distance. Once modified, a tree can be saved and revisited later. In Figure 2, PhyloViz is being used to view and search the BCL2 gene phylogenetic tree.

### Genome-wide circos viewer

**Figure 3:**
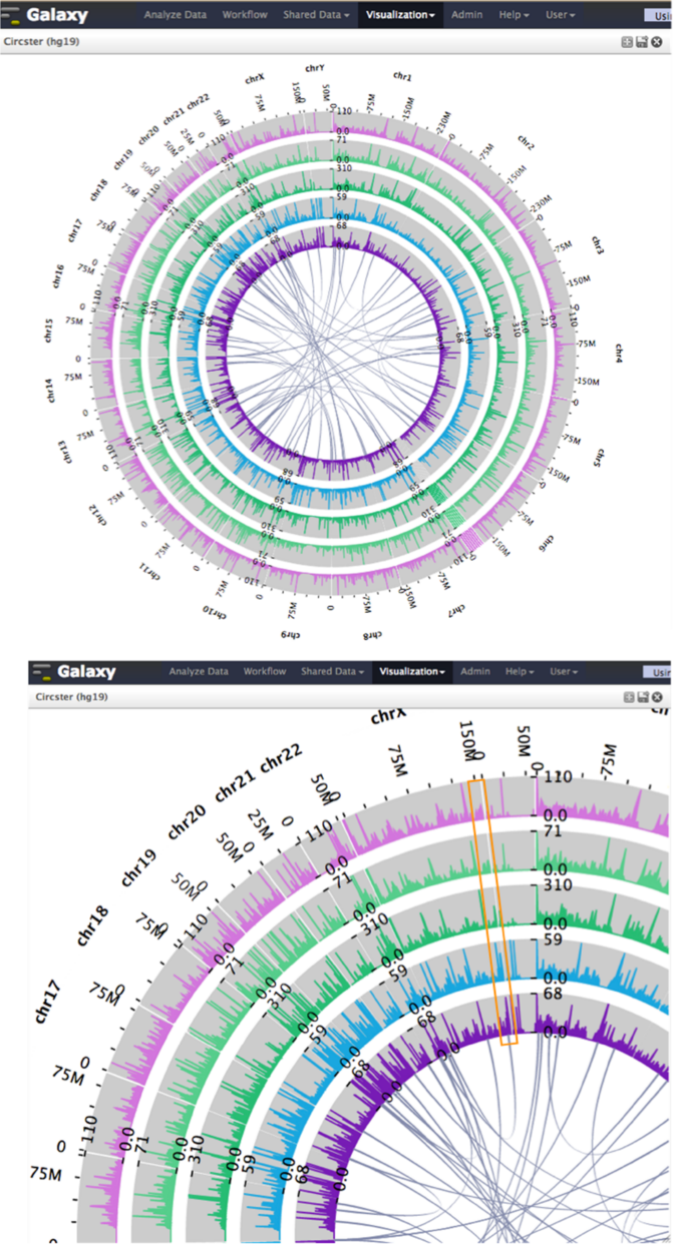
**Circster visualization of mapped RNA-seq reads and potential chimeric transcripts.** Top: from inside to out, the concentric circles in the visualization denote mapped RNA-seq reads from two bladder cancer cell lines (SRA study no. SRP013232) and mapped RNA-seq reads from three different tissue types of the BodyMap 2.0 dataset: skeletal muscle, heart, and adrenal. The arcs in the visualization represent a random selection of 50 potential chimeric transcripts from ChimerDB 2.0 Bottom: zooming in provides more details, and a region near the end of chromosome X has relatively-high expression for cancer cells but low expression for normal tissues and includes a potential chimeric transcript (orange box). The total amount of data visualized is ~20GB.

Circos visualizations [12] have become popular in genomics because they can display large, related datasets in a meaningful and aesthetic way. However, Circos plots can be difficult to create, requiring data and software downloads as well as configuration. Circster is a Circos-style viewer for rendering genome-wide data (Figure 3) on the Web. In Circster, position-based data (e.g., binding affinity, gene expression) are laid out in concentric circles representing chromosome position; chromosome interaction data (e.g., three-dimensional interactions, gene fusions) are denoted as arcs on the inside of the position data. Creating a Circster visualization and adding datasets to it is done via a graphical user interface and requires no programming experience. Datasets can be added and removed in real time, and simple animations ensure that context is preserved as changes are made to the visualization. Circster is highly interactive, enabling investigation of the data at many different levels of detail. Initially, Circster shows the complete genome, and all data is visible for all datasets. Panning and zooming around the view automatically populates it with more detailed data for visible regions.

Circster uses the D3 framework to render genomic data as SVG. Using SVG paths to display complex objects such as histograms (e.g. coverage data) and line data (e.g. conservation data) limits the number of objects created and makes the visualization scalable to a large number of datasets. Figure 3 shows a Circster visualization for mapped RNA-seq reads from both cancer cell lines and normal tissue, as well as potential chimeric transcripts from ChimerDB 2.0 [18]. Using Circster, tissue-specific expression and cancer-normal expression differences are evident near potential chimeric transcripts.

### Genome browser for high-throughput sequencing data

Genome browsers are amongst the most popular genomic visualizations, as evidenced by the large number developed (e.g., [2-4,7,19,20]) and the frequent use of browser screenshots in publications discussing genomic experiments. In genome browsers, datasets are displayed linearly along a chromosome as “tracks” and are stacked on top of each other.

**Figure 4:**
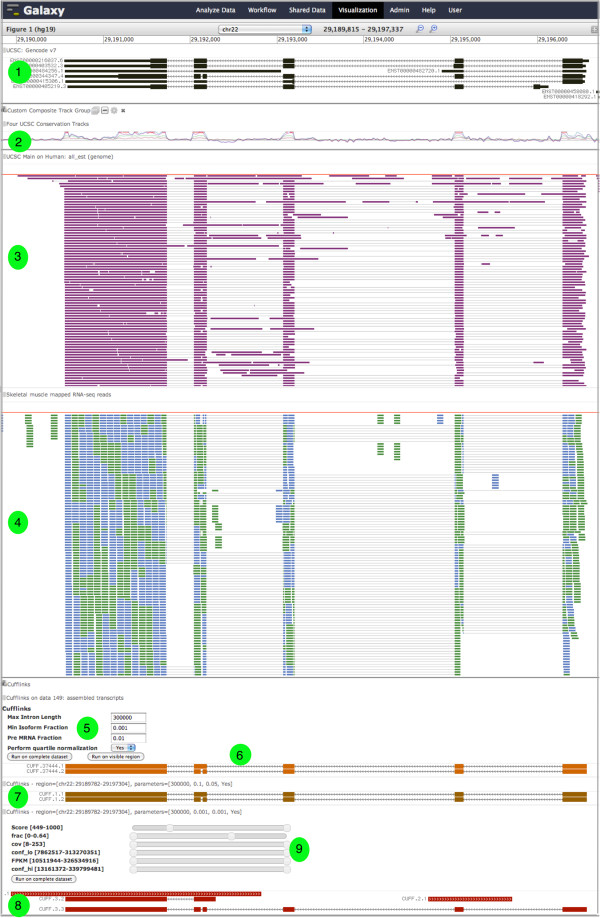
**Using Trackster to visually analyze genomic datasets on the human hg19 genome build.** The tracks and features shown include: (1) UCSC Gencode v7 gene annotation track; (2) custom rainbow track of three UCSC conservation tracks—phyloP 46-way primate conservation, phyloP 46-way mammal conservation, and phyloP 46-way vertebrate conservation; (3) UCSC all EST annotation track; (4) mapped RNA-seq reads from the skeletal muscle tissue in the BodyMap 2.0 dataset; (5) Cufflinks [13], a tool for assembling mapped reads into transcripts, has been opened as is being used to interactively assemble transcripts from the visible mapped RNA-seq reads; (6) first attempt at transcript assembly; (7-8) improving the assembly using different parameter values for Cufflinks; (9) using dynamic filters to interactively remove assembly artifacts based on attribute values.

Our genome browser, Trackster (Figure 4), is motivated by the need to enable visual exploration of increasingly large datasets produced from high-throughput sequencing data. Trackster supports all major genomic formats, including SAM/BAM, BED, GFF/GTF, VCF, Wiggle, Bedgraph, and the binary formats Bigwig and Bigbed. Using Trackster, even datasets with millions of features or mapped reads can be explored smoothly at any level of detail, from a complete chromosome of data down to individual reads. Trackster loads data asynchronously in the background so that the visualization is never refreshed, ensuring that the current viewing window and adjacent data is preserved when panning and zooming. Trackster supports smooth navigation amongst many levels of detail by using multiple data indexes, showing coverage data when there are too many features to display individually and showing individual elements when zoomed in.

Trackster customization (e.g., changing a track’s display mode or color, reordering tracks) is done interactively in the Web browser and without page refreshes, enabling rapid experimentation so that data display can be adapted to an investigation’s needs. For instance, quantitative tracks (e.g., wiggle and bedgraph) can be grouped together by drag and drop and then collapsed into a single “rainbow track” that displays all tracks in a single, dense display. Rainbow tracks are rendered instantaneously and be undone just as quickly, making it easy to try out composite tracks with different groups of tracks and settings and, ultimately, find good compositions.

Trackster is also a dedicated visual analysis environment that includes Galaxy tools for interactively filtering visualized data and even creating new tracks. The broad goal of this integration is to help users try out and see results from different tool settings, thus helping them find good settings. One common operation is filtering out unwanted data. To help find appropriate filter settings, Trackster has dynamic filters that can be used to interactively show and hide data based on feature attribute values—scores for genomic features, feature attribute values (in GFF/GTF datasets), and mapping quality scores for mapped reads.

Trackster provides a general framework for using Galaxy tools to create new tracks. Tracks visualizing data created from an analysis tool include a panel that can be used to change parameter settings and rerun the tool on the visible data to generate a new track. By repeatedly changing settings and running a tool to create new tracks, it is simple to see how particular settings influence the tool’s output and also helps identify good parameter settings. To ensure that visual analysis is fast, filters are tools are run only on visible data by default. Once good settings are found, the tool can be run with the chosen settings on complete datasets and the output placed in Galaxy’s analysis workspace.

### Tool parameter space explorer

**Figure 5:**
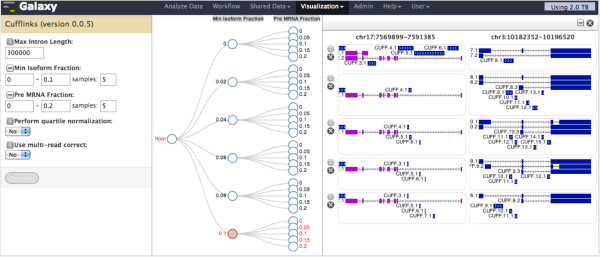
**Using Sweepster to find parameter settings that are good for assembling isoforms of tumor suppressor genes.** Isoforms are being assembled from mapped RNA-seq reads of bladder cancer cell line T24 (SRA accession no. SRX148575). An enhanced Galaxy tool form for Cufflinks (left panel), a transcript assembly tool [13], is used to create a partial tree of the tool’s parameter space (middle panel). The tree shows the parameter space for two parameters, minimum_isoform_fraction and pre-mRNA_fraction. Minimum, maximum, and number of samples are used to customize the tree. Clicking on the interior tree node where minimum_isoform_fraction equals 0.1 launches jobs for Cufflinks that systematically sample pre-mRNA_fraction from 0 to 0.2; one job is run for each value of pre-mRNA. Each track shows transcript assemblies for tumor suppressor genes TP53 (chr17:7569899-7591385) and VHL (chr3:10182352-10196520) produced by Cufflinks for a particular set of parameters (right panel). TP53 isoforms are similar across the assemblies, but VHL isoforms are substantially different. Sweepster’s tracks can be used to visually compare assemblies produced from different parameter settings and choose the settings that yield the best assembly. Once good settings are found, Cufflinks can be run on the complete dataset using the track’s controls.

While Trackster is useful for running an analysis tool to obtain data for particular parameter settings, it is not possible to see the tool parameter space or perform parameter sweeps. To address these limitations, we created Sweepster, a tool parameter space exploration application (Figure 5). Using Sweepster, a partial or complete parameter space tree for a tool is created by selecting and sampling from the tool’s inputs. Clicking on a node in the tree runs the tool iteratively to perform a systematic parameter sweep over the parameter values defined by the node and children. The tool is run on a dataset for one or more selected genomic regions, and the tool’s output for each region is visualized. Sweepster is launched via track icons in Trackster, and selected regions can include the current view in Trackster as well as bookmarked regions.

Sweepster is a combination of three complementary components: (1) an augmented Galaxy tool form; (2) the tool’s parameter space tree; and (3) track display of selected regions for each tool run. The augmented Galaxy tool form includes icons next to each parameter that add it or remove it from the tree. When a parameter is not in the tree, its value can be set to a single value using the form. For a parameter in the tree, sampling from numerical inputs is done by specifying minimum, maximum, and number of samples in the range. For categorical parameters, all possible values are included in the tree. The tool’s parameter space tree updates automatically as changes to the tool form are made. Tree levels are labeled by parameter name, and tree nodes are labeled with parameter values.

When a node is clicked in the parameter space tree, the sets of parameters denoted by the node are used to run the tool repeatedly. The output from each tool run is drawn as a set of track tiles, with each tile denoting a selected region. As in genome browsers, tracks are stacked vertically, allowing for simple comparison between regions within a track and within the same region across tracks. Mousing over a track shows its path in the parameter tree, making it easy to see the settings used to generate each track. Viewing track tiles together like this makes it simple to visually identify good settings and run the tool using the settings on the complete dataset. Finally, a track’s settings can be used to run the tool on the complete dataset.

Both Trackster and Sweepster are visual analysis applications, tightly integrating analysis tools with visualization, using tools to generate new data which is then visualized in real time. This approach works for many, but not all, tools. We have previously discussed the strengths and weaknesses of this approach, as well as applied Trackster and Sweepster to develop an RNA-seq pipeline to characterize expression dynamics of XBP1 in humans; XBP1 is a highly-conserved gene whose isoform expression is difficult to study because it includes transcripts with multiple overlapping reading frames [21].

### Using, sharing, and publishing visualizations

Icons and links in the Galaxy user interface can be used to create visualizations as well as toggle between running analysis tools and visualization/visual analysis. The history panel on the right side of Galaxy’s analysis workspace lists the datasets in a user’s current history. For each dataset that can be visualized, a icon and dynamic menu provides access to all suitable visualization applications. Some visualizations, such as the scatterplot, display in the analysis workspace, while others display on their own page. All applications include a header that provides a link back to the analysis workspace. Visual applications, including its state and preferences, can be saved and viewed or modified later.

Visualizations are first-class objects in Galaxy, and hence they can be shared, published, and included in Galaxy Pages. Visualizations can be shared with individual colleagues or a unique URL can be created for sharing more widely. Visualizations can also be published to a publically-searchable list so that they are broadly available. Galaxy Pages are interactive Web pages that include dynamic, embedded Galaxy objects—datasets, analysis histories, workflows, and now visualizations—and can be used to describe an entire genomics experiment or act an online supplement to a published manuscript. The Page at http://usegalaxy.org/interactive-rnaseq describes a recent RNA-seq experiment and includes embedded Trackster visualizations.

### Towards greater coupling of visualization and analysis tools

One way to categorize visualization applications is the degree of coupling between visualization and analysis tools. The visual applications discussed previously use widely varying levels of visualization and tool coupling. Scatterplot, Phyloviz, and Circster are loosely coupled to tools; these applications do not incorporate tools but are connected to Galaxy tools via Galaxy’s main analysis workspace. Trackster represents moderate coupling as tools are optional components of the application. In Sweepster, tools and visualization are completely coupled and both are essential for using Sweepster.

Taken together, these applications demonstrate how our framework supports visual applications regardless of their coupling with tools. However, the most powerful examples of visual analysis arise from tight coupling of visualization and tools. The key technical advance in our framework that enables tight coupling is automatic subsetting of data (including storing data subsets for repeated use) and transparently running Galaxy tools on data subsets. Data subsetting ensures that tools run quickly and that tool output can be used for interactive visual analysis.

Looking forward, we plan to apply tight coupling between visualization and analysis tools to Galaxy’s main analysis interface and its workflow system. This approach will enable more flexible and more powerful visual analysis. Currently, individual tools can be run in visualizations, but it is difficult to switch between tools. Providing access to Galaxy’s complete set of tools within a visual application would make multi-step visual analyses much easier to do. For instance, the datasets in a Galaxy history could be viewed as tracks in Trackster, and any tool could be selected and used to generate a new track. Similarly, using visual analysis tools within and alongside workflows would significantly increase the usefulness of Galaxy visual applications.

### Galaxy as a platform for visual analysis

By making it possible to create and integrate visual applications into Galaxy, we have transformed Galaxy into a platform for visual analysis of high-throughput genomic data. Galaxy provides a place where analysis tools and Web-based visual applications can be integrated and used together for visual analysis. Once a tool or visual application is integrated into Galaxy, it can be used repeatedly and in any context. Providing a single platform for analysis tools and visual applications is advantageous because tools and visual application can be combined in nearly limitless ways, ensuring that Galaxy can be used for a wide variety of genomic experiments and adapted to new experiments as well. Galaxy, then, amplifies the value of individual tools and visual applications because they can be connected with other tools and applications to create complex analyses. Creating new visual analyses is especially important as high-throughput sequencing data is increasingly being used in a wide variety of biomedical experiments, each of which may require a unique analysis pipeline.

## Conclusions

Visualization and visual analysis are important tools in high-throughput genomics experiments. Web-based visualization/visual analysis is attractive because large datasets do not need to be downloaded and because a Web browser provides common software for sharing visualizations. However, building Web-based visual applications is difficult and there are few tools to help.

We have created a framework with both client-side and server-side components that simplifies the development of Web-based visual applications. We have also integrated this framework into Galaxy and transformed Galaxy into a visual analysis platform. By enabling visual applications to be integrated into Galaxy, it is now possible to use analysis tools and visualizations together—often simultaneously—to do visual analyses of high-throughput genomic data. Using our framework, we have created five visual applications—Scatterplot, PhyloViz, Circster, Trackster, and Sweepster—and integrated them into Galaxy. These applications represent a wide variety of visualization paradigms and demonstrate the breadth of tool-visualization integration that can be achieved using our framework. In addition, these applications showcase how highly interactive and scalable Web-based visual applications can be built using modern Web technologies such as HTML5, Canvas, and D3. Every visualization can be saved, shared with colleagues, or published to the Web. As is the case for all Galaxy functionality, only a modern Web browser is required to create, view, and use Galaxy visual applications.

## Availability and requirements

**Project name:** Galaxy Visualization Framework

**Project home page:**http://galaxyproject.org

**Code home page:** All framework code and code for specific visualizations is available in the main Galaxy repository at http://bitbucket.org/galaxy/galaxy-central/

**Operating system(s):** UNIX (Solaris recommended), Linux (Ubuntu or Debian recommended), MacOS (10.6+ recommended)

**Programming language:** Python, JavaScript

**License:** Academic Free

**Any restrictions to use by non-academics:** None

## Competing interests

The authors declare that they have no competing interests.

## Authors’ contributions

JG, AN, and JT conceived the project. JG, CE, TT, AN, and JT implemented the visualization framework. CE designed and implemented Scatterplot. TT designed and implemented PhyloViz. JG and JT designed and implemented Trackster, Circster, and Sweepster. JG, CE, TT, AN, and JT wrote the manuscript. All authors read and approved the final manuscript.
